# Common and uncommon intracranial arterial anatomic variations in multi-detector computed tomography angiography (MDCTA). What radiologists should be aware of

**DOI:** 10.1007/s13244-014-0381-x

**Published:** 2015-02-14

**Authors:** Petros Zampakis, Vasilios Panagiotopoulos, Theodore Petsas, Christina Kalogeropoulou

**Affiliations:** 1 Department of Radiology, University Hospital of Patras, Rion, Greece PC 26500; 2 Department of Neurosurgery, University Hospital of Patras, Rion, Greece PC 26500

**Keywords:** Embryology, Anatomic variation, Multi-detector computed tomography, Cerebral angiography, Cerebral arteries

## Abstract

**Objectives:**

The aim of this retrospective study was twofold: (1) to show the role of multi-detector computed tomography angiography (MDCTA) in the evaluation of intracranial arterial anatomic variations; (2) to highlight their clinical importance with illustrated example cases.

**Materials and methods:**

One thousand seven hundred thirty-nine patients who underwent carotid and/or cerebral CTA using a 16-row multi-detector CT over the last 9 years were retrospectively analysed with attention to the presence of persistent carotid-basilar anastomosis and other intracranial arterial variations.

**Results:**

All kinds of persistent carotid-basilar anastomosis were present in our series. The most common was the presence of fetal pCom (23 %). From the other studied anatomic variants, the most common was the presence of a hypoplastic A1 segment. In all cases CTA was an excellent diagnostic tool, providing not only high-resolution angiographic images, but also details of the surrounding structures.

**Conclusions:**

The knowledge of intracranial anatomic variations could be very important for the treatment planning of patients who need neurointervention or to explain uncommon and unexpected clinical findings. CTA can reliably provide this kind of information by depicting intracranial anatomic variations.

**Teaching Points:**

• *Knowledge of intracranial anatomic variations is important.*

• *Radiologist should be aware of the intracranial anatomic variations.*

• *Computed tomography angiography can reliably depict intracranial anatomic variations.*

## Introduction

The existence of persistent embryonic communications between the carotid and vertebro-basilar arterial systems and other anatomic variations is well recognised. As endovascular procedures become even more common, the need for detailed understanding of such anatomy, together with the variations that may be encountered becomes more pressing. These anatomical variations reflect the embryological development of the organism and the phylogeny of the species. Through study of embryology and evolution, the element of time is emphasised and thus the realisation of the anatomy as a four-dimensional subject [1].

Over the last years, the developments in technology regarding modern imaging have established computed tomography angiography (CTA) as an essential tool for the study of intracranial vasculature. CTA provides not only high-resolution angiographic images but also excellent details of the surrounding structures as well [2]. This can be very important for the recognition of certain anatomic variations. In our department we perform MDCTA as a routine examination for the study of intracranial or cervical vessels as the first and many times only imaging technique, when indicated.

We pictorially reviewed not only the anatomic configuration of the carotid-vertebrobasilar anastomoses, but also some other rare anatomic variations as they appear in the CTA and illustrate their importance in clinical practice.

## Materials and methods

We retrospectively analysed 1,739 patients who were scanned in our department because of potential carotid or intracerebral vascular pathology over the last 9 years (September 2005 and June 2014). The main indications were symptomatic or asymptomatic carotid disease, acute stroke, persistent headaches, intracranial (subarachnoid, intracerebral or intraventricular) haemorrhage and CNS vasculitis.

All patients, depending on their clinical indication, underwent a carotid and/or cerebral computed tomography angiography (CTA) using a 16-row multi-detector CT (Lightspeed 500, GE) (Waukesha, WI, USA) as a routine examination.

### Carotid CTA protocol

Patients were placed in a supine position with neck hyperextension. Images were obtained from the level of the aortic arch, including the entire cranial cavity. Following removal of prosthetic teeth, 120 ml contrast media was administrated in all cases with an automatic injector at a rate of 3.5 ml/s. The region of interest was positioned at the aortic arch, and the threshold for CT angiography was set as 100 HU. When the threshold was surpassed, helical imaging was automatically initiated. Slice thickness was 1.25 mm and the feed 0.625 mm.

### Intracranial CTA protocol

The patient was placed in a supine position. Images were obtained from the level of the skull base, including the entire cranial cavity; 120 ml contrast media was administrated in all cases with an automatic injector at a rate of 3.5 ml/s. The region of interest was positioned at the upper cervical area (C3 level), and the threshold for CT angiography was set as 100 HU. When the threshold was surpassed, helical scanning was automatically initiated. Slice thickness was 0.625 mm and the feed 0.625 mm.

Raw data were analysed using a standard workstation and three-level reconstruction (MPR) and 3D algorithms were applied.

All CTAs were reviewed by an experienced neuroradiologist (P.Z.) with a main interest in vascular cervical/ intracranial anatomy and an experienced radiologist (C.K.) with attention to the presence of persistent carotid-basilar anastomosis and other intracranial arterial variations such as fenestrations, hypoplasia, aplasia or other ACA-MCA variants. The reviewers came to a consensus decision regarding the findings.

## Results

### Persistent carotid-basilar anastomosis

**Fig. 1:**
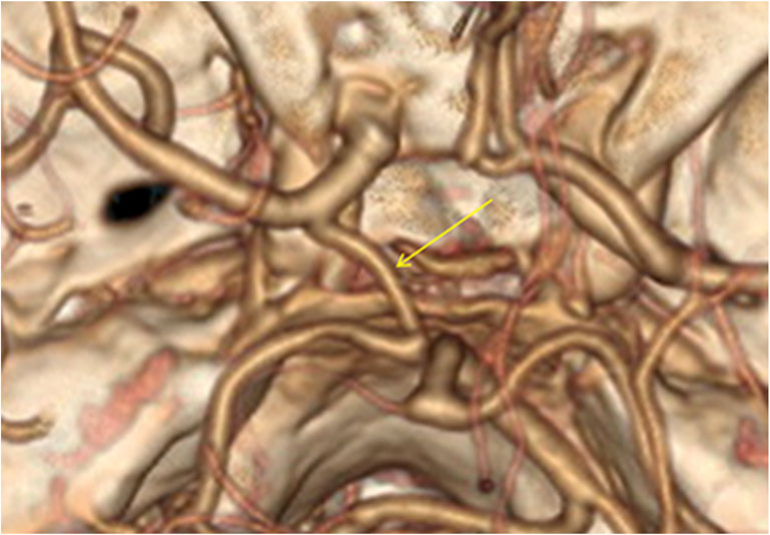
CTA (VRT 3D reconstructions) shows a left-sided foetal Pcom (*yellow arrow*)

**Fig. 2:**
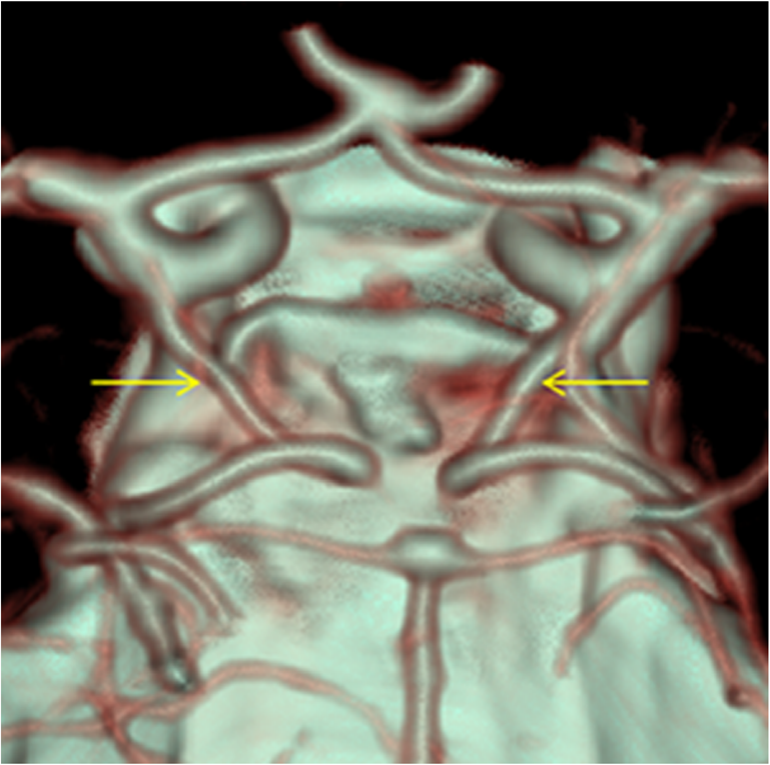
CTA (VRT 3D reconstructions) shows a bilateral foetal Pcom (*yellow arrows*)

The most common persistent carotid-basilar anastomosis was the presence of a foetal type of posterior communicating artery. This variation was found in 399 patients (23 %), 209 cases on the left and 113 on the right, while a bilateral location was present in 77 patients (Figs. 1 and 2).

**Fig. 3:**
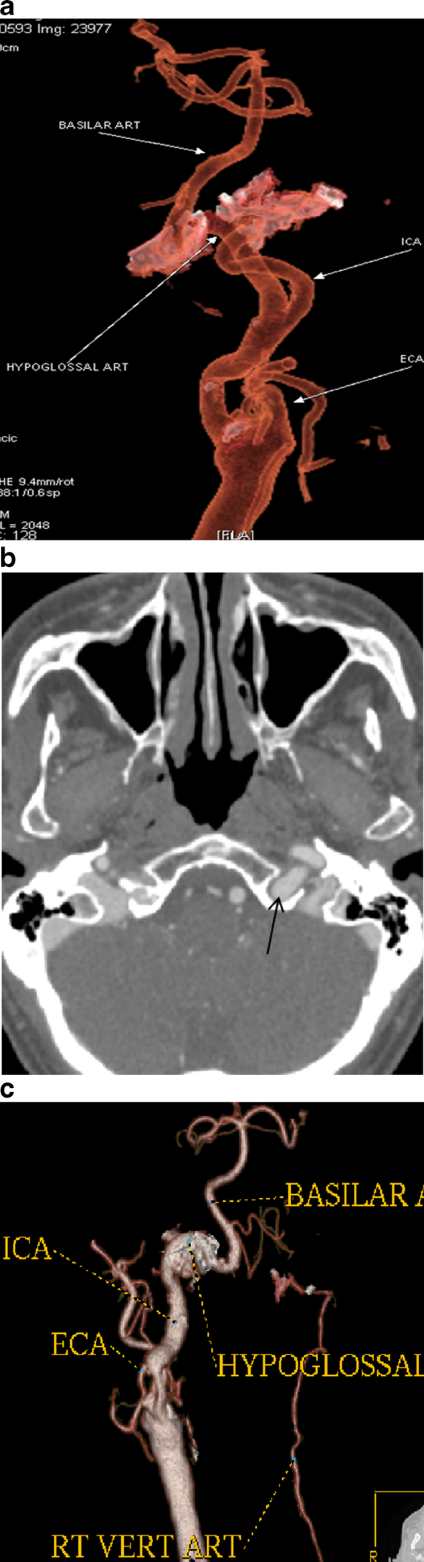
**a** CTA (VRT 3D reconstructions) shows a vessel connecting the ICA with the basilar artery (annotations). **b** Axial CT image at the level of the hypoglossal canal shows an enlarged vessel piercing the skull base on the left, through the hypoglassal canal, which was also enlarged (*black arrow*). **c** CTA (VRT 3D reconstructions) reveals an absent proximal vertebral artery and hypoplastic contralateral vertebral artery (annotations)

**Fig. 4:**
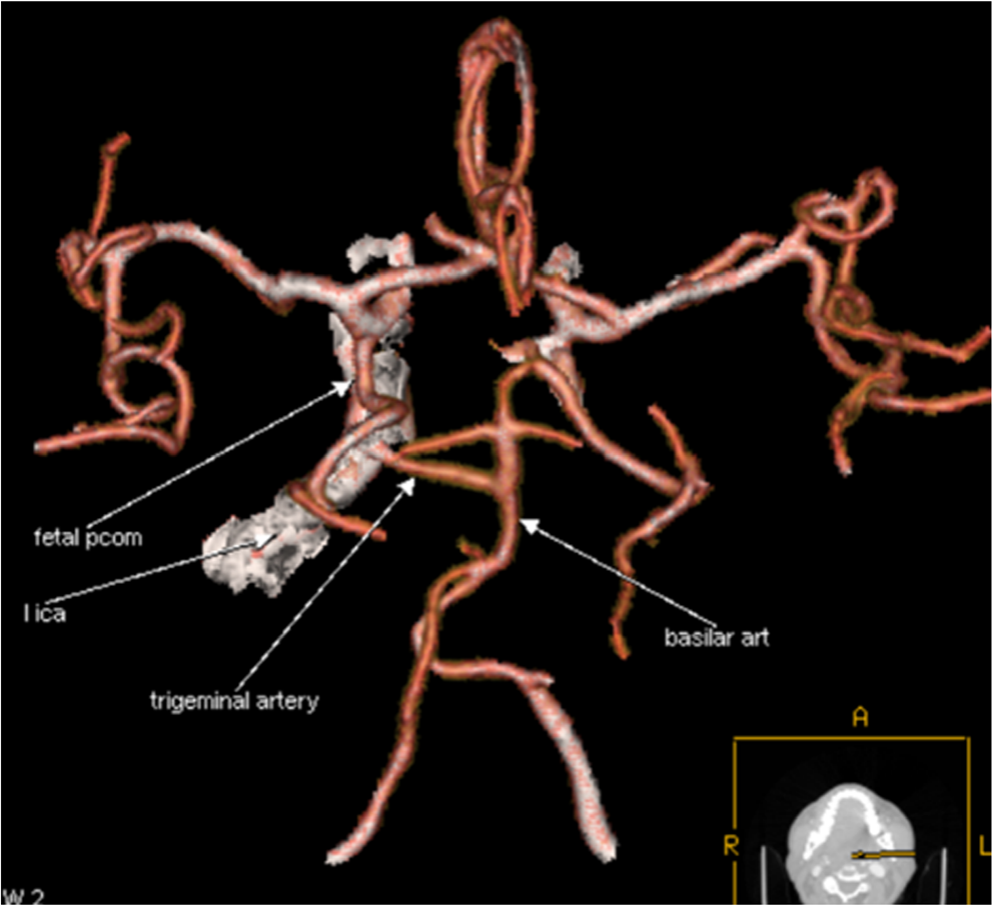
CTA (VRT 3D reconstructions) shows a left-sided trigeminal artery joining the basilar artery. The basilar artery (which is relatively hypoplastic, proximal to the connection site) gives rise to the right PCA, while the left PCA arises from the ICA (foetal PCom) (Saltzman’s type II or Weon’s type 3) (annotations)

**Fig. 5:**
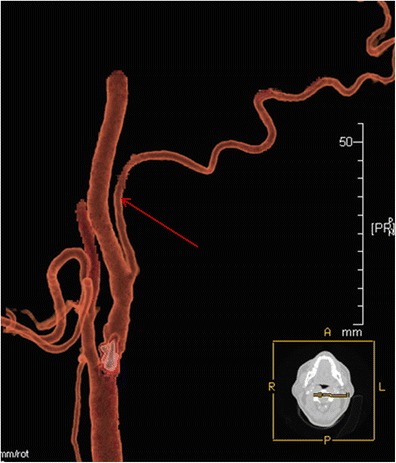
CTA (VRT 3D reconstructions) shows a case of the origin of the occipital artery from the ICA (*red arrow*)

A persistent hypoglossal artery was seen in one case (0.057 %) (Fig. 3a–c), a Saltzman type 2 persistent trigeminal artery was found in one patient (0.057 %) (Fig. 4), while a variation of a type 1 proatlantal intersegmental artery was present in two (ICA origin of the occipital artery) (0.115 %; Fig. 5).

### Other intracranial arterial variants

**Fig. 6:**
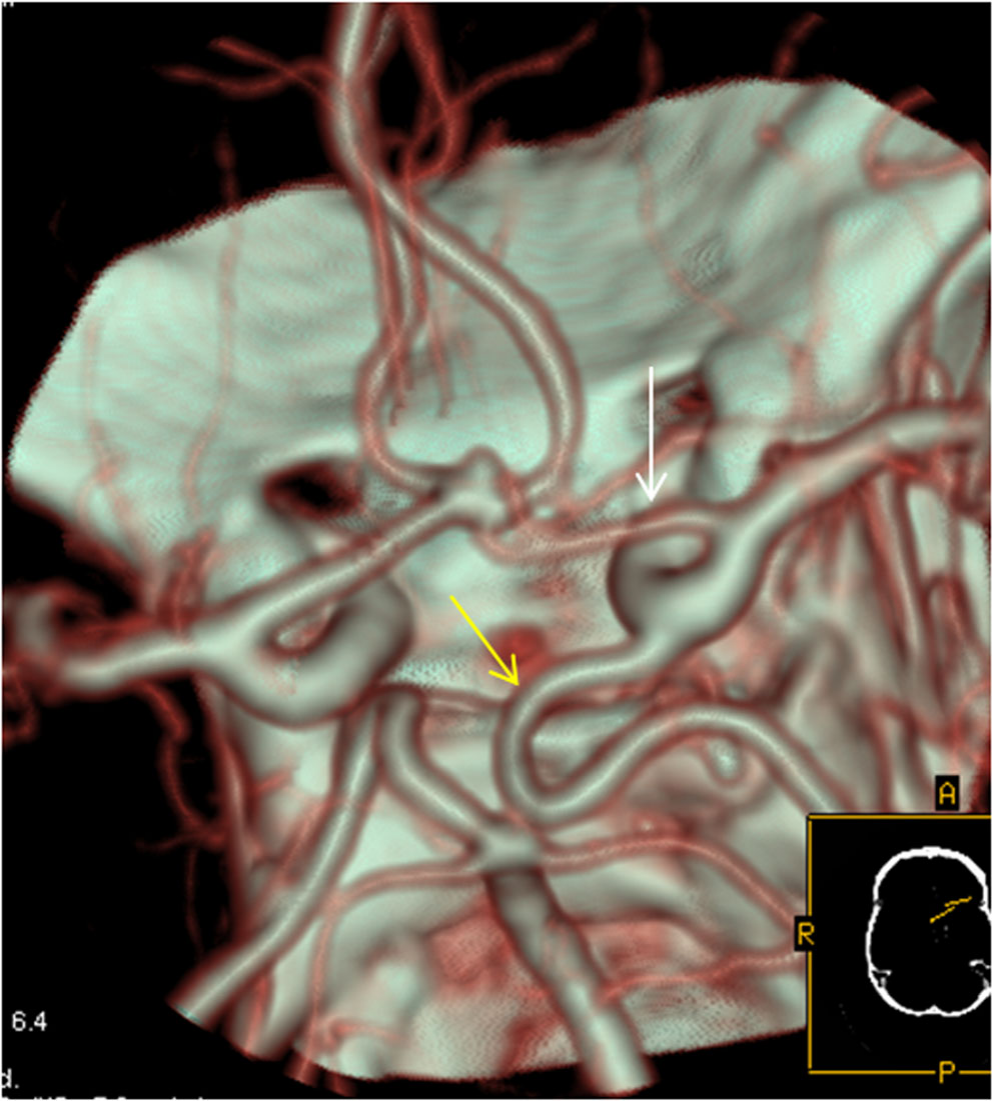
CTA (VRT 3D reconstructions) shows a hypoplastic right-sided A1 segment of the anterior cerebral artery (*white arrow*) in a patient with an Acom aneurysm. Note the presence of a foetal Pcom on the same side (*yellow arrow*)

**Fig. 7:**
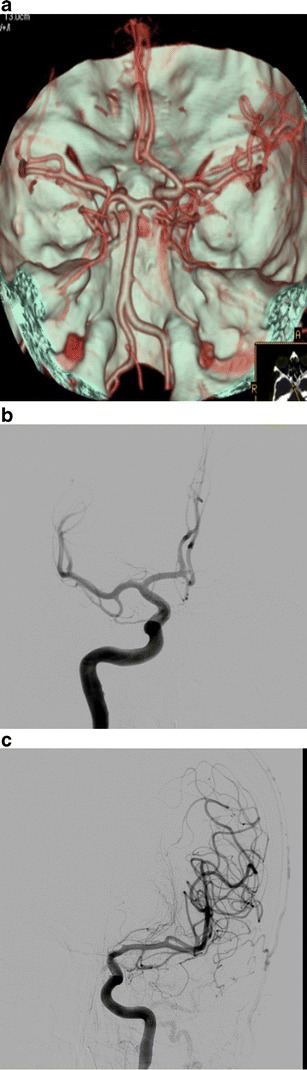
**a** CTA (VRT 3D reconstructions) reveals aplasia of the left A1 segment of the anterior cerebral artery in a patient with an Acom aneurysm. **b** Digital subtraction angiography (*AP view*) of the right ICA verifies the presence of a small Acom aneurysm. Both A2 segments are opacified from this single injection. **c** Digital subtraction angiography (*AP view*) of the left ICA. The left A1 segment is absent

The most common variant was the presence of a hypoplastic A1 segment of the anterior cerebral artery (ACA) (Fig. 6). This was observed in 27 % of the patients (*n* = 469). Aplasia of A1 was seen in 9 % of the patients (*n* = 156), and this was confirmed in all cases where the patients underwent DSA for diagnostic or therapeutic reasons (Fig. 7a–c). Another common variant was the hypoplastic V4 segment of the vertebral artery, which was seen in 87 individuals (5 %), whereas in 32 cases (1.8 %), the segment was completely or partially aplastic (the vertebral artery terminating as a posterior inferior cerebellar artery).

**Fig. 8:**
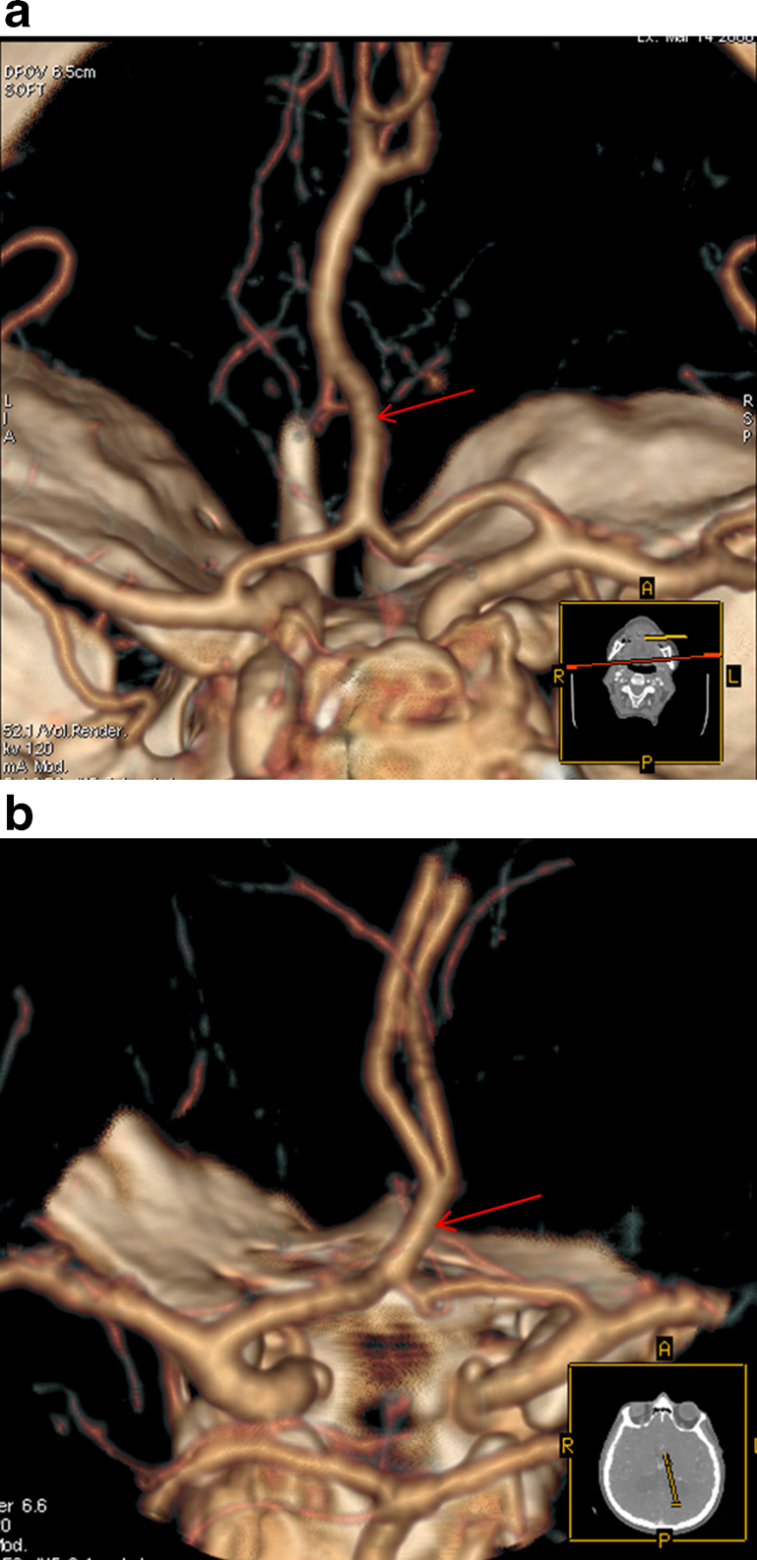
**a** CTA (VRT 3D reconstructions) reveals the presence of an unpaired ACA (conventional type, long segment) (*red arrow*). **b** CTA (VRT 3D reconstructions) shows an unpaired ACA (conventional type, short segment) (*red arrow*)

**Fig. 9:**
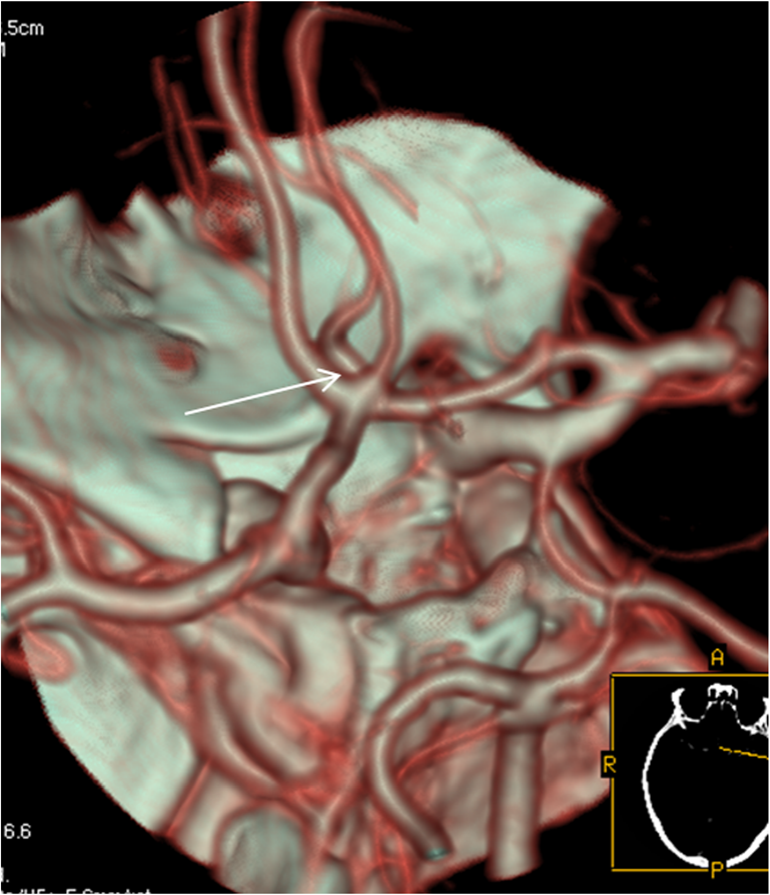
CTA (VRT 3D reconstructions) reveals the presence of a triplicated ACA (*white arrow*)

The percentage of unpaired anterior cerebral arteries (ACA) was 0.4 % (7 patients) (Fig. 8a–b). In this variation there is a common arterial trunk arrangement that supplies both hemispheres. ACA trifurcation was seen in 1.5 % of the patients (*n* = 26) (Fig. 9).

**Fig. 10:**
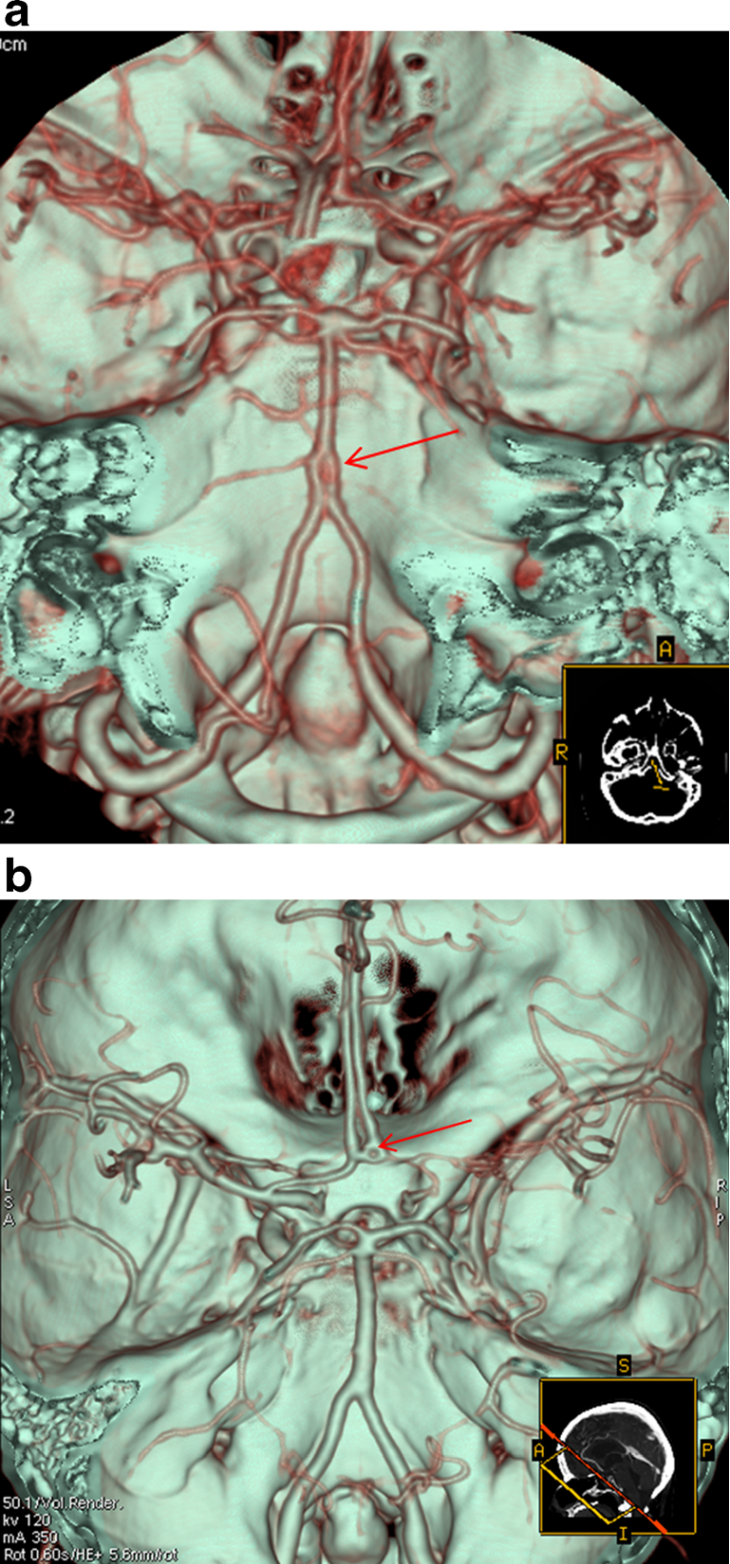
**a** CTA (VRT 3D reconstructions) shows a proximal basilar artery fenestration (*red arrow*). **b** CTA (VRT 3D reconstructions) shows an Acom artery fenestration (*red arrow*)

Intracranial arterial fenestrations were found in 17 (∼1 %) patients. The most common location was the vertebrobasilar system, with 11 basilar artery fenestrations. The remaining fenestrations were found at the anterior communicating artery (AComA) (Fig. 10a and b).

**Fig. 11:**
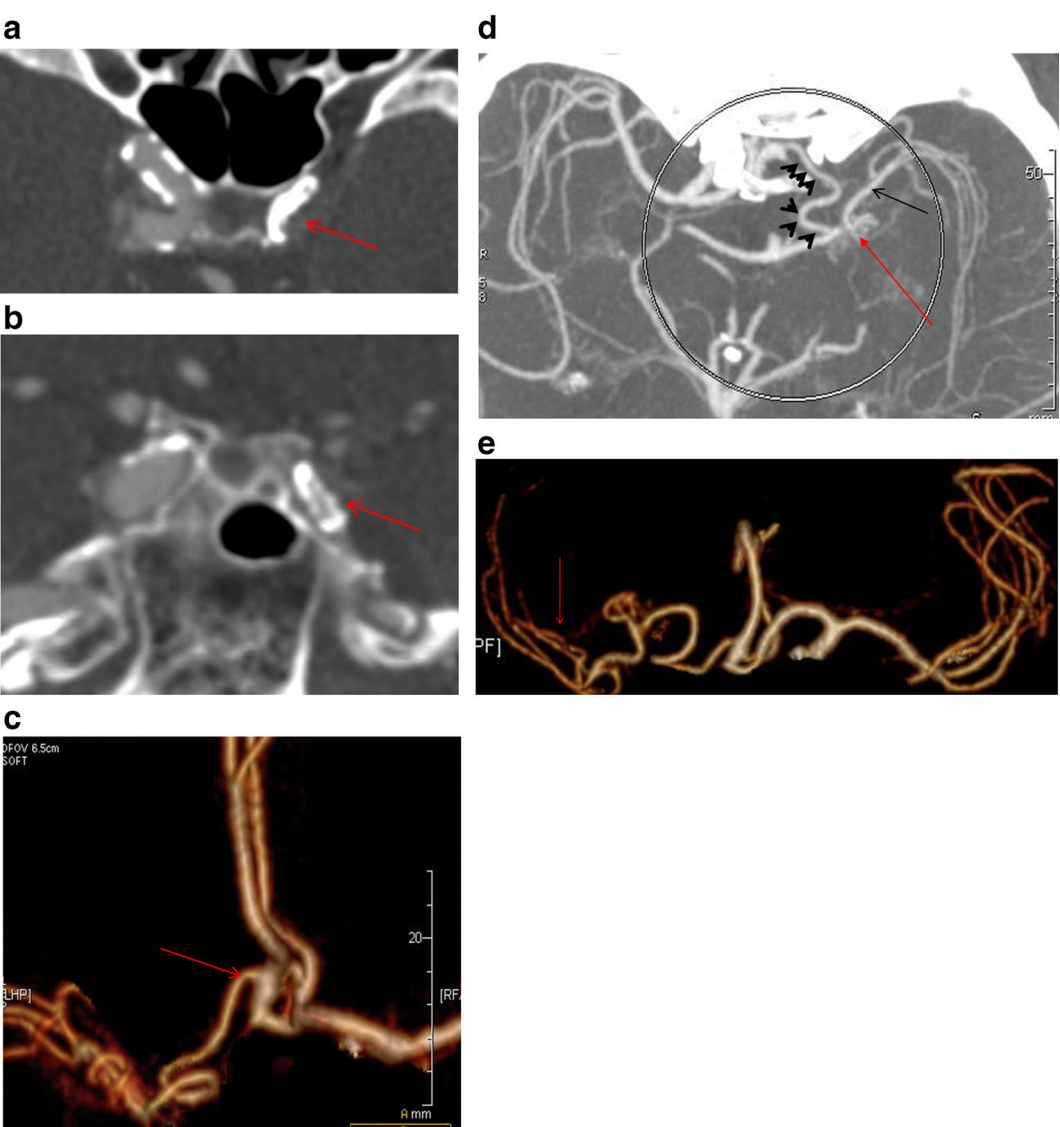
**a** Axial CT image at the level of the cavernous carotids shows occlusion of the left ICA (*red arrow*) due to atherosclerotic disease. **b** Coronal reconstruction at the level of the cavernous carotids verifies the occlusion of the left ICA (*red arrow*) as well as atherosclerotic disease. **c** CTA (VRT 3D reconstructions) shows the accessory MCA as a vessel coming from the A2 segment of the ipsilateral anterior cerebral artery (*red arrow*). **d** Axial MIP image shows the course of the AccMCA (*black arrowheads*), the anastomotic network (moyamoya type) at the level of the left mid M1 segment (*red arrow*) as well as the patent peripheral left MCA (*black arrow*). **e** CTA (VRT 3D reconstructions) reveals the same configuration and patent peripheral MCA (*red arrow*)

Finally we encountered the presence of an accessory middle cerebral artery in one patient with occlusive carotid disease (Fig. 11a–e).

## Discussion

Over the last 10 years, multi-detector computed tomography angiography (MDCTA) has become a first line tool for the evaluation of the cervical and intracerebral arterial vasculature [2]. In the majority of referral centres, it is the first and most often only examination for the detection of brain arterial pathology. It provides high-quality angiographic images as well as information for the surrounding anatomic structures. It is also very helpful for the mapping of the arterial tree in cases of endovascular or surgical intervention. Anatomic variants of the intracranial circulation are frequent incidental finding on CTAs [3–7]. Although most of these variations are of minor clinical importance, some may play a significant clinical role and their recognition on CTA examinations is important for surgical and endovascular treatment planning.

We will describe the embryology and importance of the most clinically significant variants.

### Embryology of carotid-basilar anastomosis

On a 4-mm embryo (approximately 30-day gestation), the perineural vascular plexus extends over the neural tube. On the ventral surface of the tube, this formed the two paramedian longitudinal neural arteries (LNAs) at the hindbrain. The ventral aorta, emerging from the heart, supplies the aortic arches. The first two arches have already regressed while the third arch and cephalic end of the dorsal aorta are gradually developing into the internal carotid artery. This carotid system directly supplies the forebrain. The LNAs (precursors to the basilar artery) supply the developing hindbrain and receive blood from the carotid and dorsal aortas via a series of transient anastomoses: at the level of the first cervical vertebra is the proatlantal artery, followed by the hypoglossal artery (traversing the hypoglossal canal) and trigeminal artery at the level of the trigeminal nerve. These vessels appear and regress in succession and are not present at the same time. The vertebral artery has not yet formed while the posterior communicating (PCom) artery is developing.

All of these occur between the 5–12-mm embryo stage.

In the 12–14-mm embryo, fusion of the PCom artery with the cranial end of the LNA coincides with regression of the trigeminal artery. Flow in the two LNAs, which progressively fuse to form the basilar artery, is thus from cranial to caudal.

The vertebral arteries develop from intersegmental anastomoses between the cervical segmental arteries and their fusion with the caudal end of the hindbrain LNA leads to rapid regression of the PCom arteries. This causes flow reversal in the developing LNA/basilar system, establishing the adult circulation pattern by the time the embryo is 15 mm [1, 8].

### Persistent posterior communicating artery

**Fig. 12:**
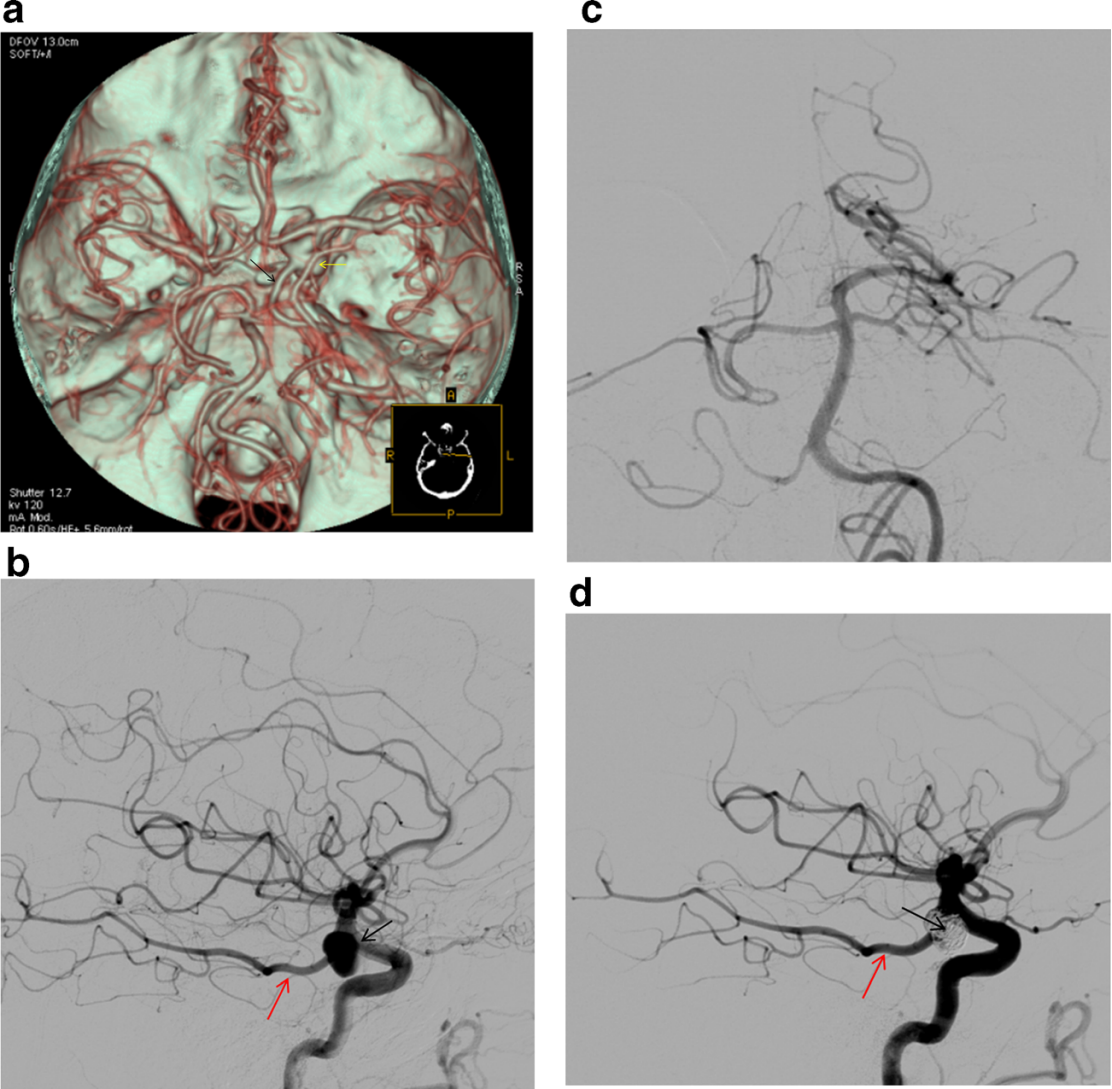
**a** CTA (VRT reconstructions) shows a right-sided foetal Pcom (*black arrow*) with a large aneurysm at the origin of the vessel (*yellow arrow*). **b** Digital subtraction angiography (*lateral view*) of the right ICA shows a foetal Pcom (*red arrow*) and large aneurysm at the origin of the vessel (*black arrow*), which cannot be compromised. **c** Digital subtraction angiography (*AP view*) of the left vertebral artery. The posterior cerebral artery on the right is not opacified (definition of a foetal Pcom). **b** Post-embolisation digital subtraction angiography (*lateral view*) of the right ICA shows good patency of the foetal Pcom (*red arrow*) and complete obliteration of the aneurysmal sac (*black arrow*)

Following the development of the vertebral artery and establishment of the typical adult posterior circulation, persistence of one of the transient foetal anastomoses may be seen. The PCom normally remains a small vessel. If the PCom fails to regress and persists as the main supply to the posterior cerebral artery, it is termed a foetal type PCom. It is a very common configuration (20–30 % of anatomic specimens) and may be unilateral or bilateral. The CTA appearance of this variation includes the origin of the posterior cerebral artery from the ICA as well as the absence of the ipsilateral P1 segment of the posterior cerebral artery connecting to the basilar artery. In our series the percentage of patients having this common variation was 23 %, in accordance with the spectrum of prevalence (10–37 %) of other published studies [3, 7]. The usefulness of the knowledge of this variation is twofold. On the one hand, it is very important when treating patients with Pcom aneurysms in which the vessel comes out of the aneurysmal sac. It is crucial to be aware of the existence of this variation in order to decide whether you can sacrifice the pCom or not. In our institute all patients with Pcom aneurysms and presence of a foetal pcom from the CTA are carefully examined with digital angiography prior to any endovascular procedure (Fig. 12a–d).

**Fig. 13:**
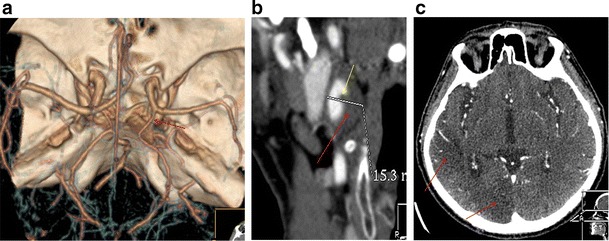
**a** CTA (VRT reconstructions) shows a right-sided foetal Pcom (*red arrow*). **b** CT oblique MPR image shows an increased wall thickness (*red arrow*) and intimal flap (*yellow arrow*) of the dissected right ICA. **c** Axial CT image at the level of the third ventricle shows right-sided occipital and posterior parietal lobe infarcts (*red arrows*)

Another point of clinical significance is that carotid pathology may cause a “posterior circulation” infarct, as shown in a patient with a foetal Pcom and dissection of the ipsilateral carotid (Fig. 13a–c).

### Persistent trigeminal artery

This is the most common of the remaining persistent foetal arteries. It is present in 0.1–1 % of angiograms and autopsies [9, 10]. In our series the percentage of patients having this uncommon variation was 0.057 %, lower than the reported angiographic percentage in other published CTA studies [3, 11].

There are three anatomical patterns (types) of persistent trigeminal artery according to Saltzman [12], while more recently Weon et al. [13] proposed a new classification system of four types.

The persistent primitive trigeminal artery (PPTA) arises from the cavernous carotid and joins the basilar artery on its distal part. The proximal vertebral artery is usually but not always hypoplastic. We illustrate a case of PPTA that is classified as type II according to Saltzman or type 3 according to the Weon classification. (Fig. 4). Recognition of this vessel could be important prior to Wada testing to avoid unexpected brain stem anaesthesia and is obviously important for any endovascular treatment! Some authorities have found aneurysms more commonly associated with persistent trigeminal arteries, while others have highlighted the importance of PPTA in cerebrovascular disease [14, 15].

### Persistent primitive hypoglossal artery

Rarer than the trigeminal artery, the persistent primitive hypoglossal artery (PPHA) is seen in 0.02–0.2 % of angiograms. In our series, the percentage of patients having this uncommon variation was 0.057 %, in accordance with the published studies [16, 17].

The vessel usually arises from the ICA between the C1 and C3 vertebral levels. It traverses the hypoglossal canal and joins the lower basilar artery (Fig. 3a). It does not pass through the foramen magnum, but enters the cranial cavity through the hypoglossal canal, which is enlarged (Fig. 3b). The proximal vertebral artery is usually absent and the contralateral vertebral artery is hypoplastic (Fig. 3c) [18].

Failure to recognise this vessel could be hazardous in skull-base surgery and in performing Wada testing. Even more importantly, as previously reported, the hypoglossal branch of the ascending pharyngeal artery is a remnant of the PPHA. Therefore careless particle embolisation in the ascending pharyngeal territory could result in a vertebro-basilar territory infarction. A case of this anatomic variation has been illustrated in a recent case report [17].

### Persistent proatlantal intersegmental artery

The proatlantal arteries are segmental vessels joining the caudal portion of the hindbrain LNA at the C1 and C2 levels (a type 1 proatlantal intersegmental artery corresponds to the first segmental artery, while type 2 corresponds to the second segmental artery). The vertebral arteries (VAs) form from intersegmental anastomoses between the lower cervical segmental arteries and subsequently fuse with the proatlantal artery to give the adult configuration. The C1 portion of the VA thus represents the remnant of the proatlantal artery. Full persistence of the proatlantal arteries is uncommon but normal anastomoses between the occipital artery and the VA at the C1 and C2 levels should be anticipated. These can be hazardous when embolising a dural AV fistula supplied by the occipital artery.

An ICA origin of the occipital artery is a variation of a type 1 proatlantal intersegmental artery. In our series of patients, we observed this variation in 0.115 % (Fig. 9) [in accordance with the current literature finding of 0.08–0.2 %, but lower than in a recently reported study (0.49 %)] [19].

### Accessory middle cerebral artery (AccMCA)

The variations of the AccMCA express the phylogenetic origins of the MCA from a group of vessels with similar potentials in the early stages of evolution, including the recurrent artery of Heubner (RAH). Therefore, a distal ACA origin of the AccMCA corresponds to an enlarged RAH. This was described by Manelfe in 1977 as type 3, where the AccMCA is a Heubner artery with an extensive cortical supply, arising from the proximal part of the A2 segment [20].

An AccMCA has to be differentiated from a duplicated MCA. Embryologically, an AccMCA is a vessel with a striatal supply that originates from the proximal or distal ACA, while a duplicated MCA is a vessel with a cortical supply that originates from the terminal ICA.

AccMCAs are relatively rare (0.3–4 %) [21] and have been described in relation to the presence of brain aneurysms. The most important clinical role of this variation is in cases of severe stenosis/occlusion of the proximal carotid, where this vessel is actually a natural bypass [22, 23]. This is illustrated in one of our cases (Fig. 11a–e) of an occluded intracranial proximal ICA where an AccMCA via an anastomotic network (moyamoya type) played the role of the occluded proximal MCA. The distal MCA was patent and the patient did not suffer a stroke.

### Other intracranial arterial variants (hypoplasias, aplasias, fenestrations, ACA variations)

The most common of other intracranial arterial variants was the presence of a hypoplastic A1 segment. This was observed in 27 % of our patients. Although this variation was present in the vast majority of patients with Acom aneurysms, the role of this variation in the formation of aneurysms is unclear. Proper statistical analysis must be done in order to determine whether such a relationship exists. This was not in the scope of this study.

Aplasia of A1 was seen in 9 % of our patients. This anatomic variation is of crucial importance in cases of Acom aneurysms where the neurointerventionist must be aware that possible compromise of the neck of the aneurysm (either endovascular or surgical) could lead to bilateral frontal lobe ischaemic events. The same applies for the presence of an unpaired ACA (short or long segment) [24].

Intracranial arterial fenestrations were found in 17 (∼1 %) patients. The most common location was the vertebrobasilar system, with 11 basilar artery fenestrations. The remaining fenestrations were found at the anterior communicating artery (AComA). The clinical significance of this variation is the association with the presence of an arterial aneurysm in the fenestration site. This has been addressed in a recently published paper by Patel et al. [25].

## Summary

Overall the knowledge of the embryology and anatomy of all these described anatomic variations could be very important for the surgical or endovascular treatment planning for patients who need neurointerventions or have CNS-related symptoms.

Since CT angiography has become a first line tool for the evaluation of intracranial vasculature mapping, the radiologists should be aware of these relatively rare and other more common variants in order to help neurointerventionists or explain uncommon and unexpected clinical findings.
